# Viral proteins as discovery tools for cell biology: The case of the geminivirus-encoded C4

**DOI:** 10.1371/journal.ppat.1013798

**Published:** 2026-01-05

**Authors:** Shaojun Pan, Hua Wei, Frederik Dröst, Amit Fenn, Nadia Kamal, Rosa Lozano-Durán

**Affiliations:** 1 Department of Plant Biochemistry, Center for Plant Molecular Biology (ZMBP), Eberhard Karls University, Tübingen, Germany; 2 School of Life Sciences, Technical University of Munich, Munich, Germany; CAU: Christian-Albrechts-Universitat zu Kiel, GERMANY

## Viruses are master manipulators

Viruses are obligate intracellular parasites that co-opt and utilize the molecular machinery of the cells they infect to multiply and spread. With, in most cases, only a handful of viral proteins encoded in their small DNA or RNA genomes, viruses are among the simplest biological entities. However, following their fast-paced evolution, enabled by high replication error rates and large population sizes, viruses are extremely efficient in their manipulation of the host cell. Viral proteins suppress, promote, redirect, or fine-tune cellular processes, often through physical interaction with proteins in the cell. Considering the evolutionary pressure for efficacy and specificity, viral proteins are excellent probes to study cell functions and investigate the underlying mechanisms, offering unique and powerful insights into cell and molecular biology. In fact, following virus-encoded proteins has historically shed light on biological processes as fundamental as nuclear trafficking, apoptosis, cell cycle regulation, or gene silencing.

## Plant viruses as discovery tools

Every living creature described to date can be infected by viruses. Although in all cases viruses need to subvert cell biology and hijack the available molecular machinery to mediate their “vital” functions, viral infections in different kingdoms have their idiosyncrasies. In plants, viruses need to reach a sessile host, frequently with the aid of vector organisms; suppress different layers of antiviral innate immunity; move from cell to cell through plasmodesmata; and spread throughout the organism in the absence of a circulatory system. Once all of this is accomplished, plant viral infections can lead to dramatic developmental alterations that we recognize as symptoms, which can result in significant yield losses in agriculture [1].

Uncovering the function of viral proteins in plants can help us understand fundamental plant molecular and cell biology, physiology, and even evolution. Owing to genome size constraints, possibly due to limitations imposed by cell-to-cell transport and/or encapsidation, plant viral proteins tend to be small (<45 kDa) [2,3] but multifunctional and establish multiple contacts with the host proteome through physical interaction. In addition, an ever-growing body of data indicates that plant viral proteins, similar to what has been inferred in animal-infecting viruses, tend to target host proteins that are more conserved than average and/or that are hubs—that is, highly connected proteins in the network of protein–protein interactions [4–6]. Conservation suggests functional relevance; conserved proteins frequently have central functions in the cell and are therefore assumed be less tolerant to mutations, making them strategic targets and impeding viral evasion by the host. Hub proteins, on the other hand, are more likely to play prominent regulatory roles, offering the virus broad leverage over the cell.

## Following geminiviruses to illuminate plant biology

A viral family that entails high potential as a discovery tool for plant biology is *Geminiviridae*. Geminiviruses—the members of this family—have circular, single-stranded DNA genomes that replicate in the nucleus of the infected plant cell. To enable viral DNA replication, geminiviruses need to manipulate the cell cycle, in analogy to mammalian oncoviruses, and subvert and repurpose the plant DNA replication machinery. Moreover, these viruses hijack transcription and splicing to enable viral gene expression and take over translation to produce viral proteins. Virus-encoded proteins also hitchhike the cell’s protein trafficking and post-translational modification pathways to reach their targets and manipulate cell host functions to promote infection, including the suppression of antiviral defence mechanisms. Viruses in the family *Geminiviridae* have recently provided an excellent example of how chasing virus-encoded proteins can inform infection biology and host biology in general, illustrated in a 10-kDa geminivirus-encoded protein called C4.

## The small but mighty geminiviral C4 protein

**Fig 1:**
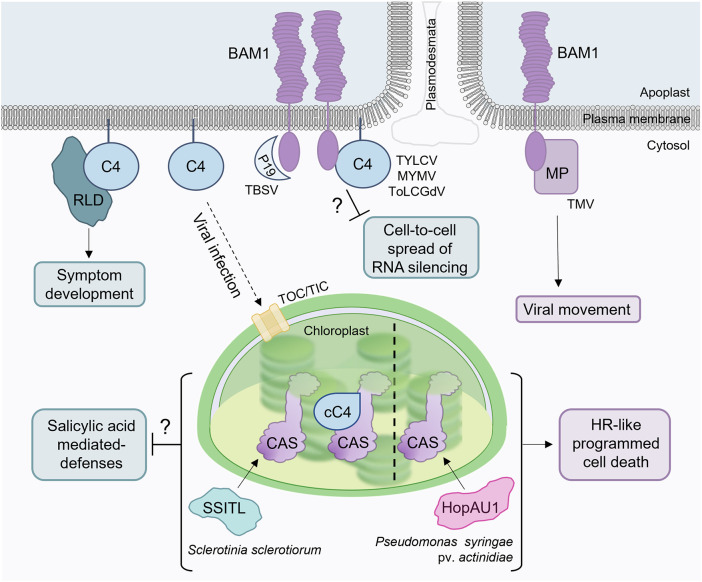
The C4 protein from the geminivirus tomato yellow leaf curl virus (TYLCV) helps shed light onto the function of its target plant proteins in defence and development. Three known interactors of C4 from TYLCV are shown: the receptor-like kinase BAM1, RLD proteins, and CAS; the potential outcome of each interaction is indicated in boxes. Arrows or blunt-end lines pointing at boxes denote activation or inhibition, respectively; dashed lines represent protein re-localization. Question marks indicate connections that are currently correlative. Other pathogen-encoded proteins convergently targeting these proteins are shown, with species names. cC4: chloroplastic C4 (after removal of the chloroplast transit peptide following chloroplast import). TBSV: tomato bushy stunt virus. MYMV: mungbean yellow mosaic virus. ToLCGdV: tomato leaf curl Guangdong virus. TMV, tobacco mosaic virus; MP, movement protein; HR, hypersensitive response. The plasma membrane icon is from http://www.clker.com.

C4 was, until recently, considered the smallest protein encoded by geminiviruses. This protein is the most divergent among the canonical geminiviral proteins and has been ascribed a diversity of functions across species [7–9]. Work on the C4 protein from tomato yellow leaf curl virus (TYLCV) showed that this protein can suppress the cell-to-cell movement of RNA silencing [10,11], a function hypothesized to be mediated by its physical interaction with the receptor kinase BARELY ANY MERISTEM 1 (BAM1) at the plasma membrane and, in particular, at plasmodesmata [11] (Fig 1). Both function and interaction seem to be conserved in other geminivirus species, as demonstrated for mungbean yellow mosaic virus (MYMV) and tomato leaf curl Guangdong virus (ToLCGdV) [12,13]. Interestingly, BAM1 is also targeted at plasmodesmata by evolutionarily unrelated viral proteins, namely the silencing suppressor P19 from tomato bushy stunt virus (TBSV) and the movement protein (MP) from tobacco mosaic virus (TMV) [14,15], suggesting that this receptor might be a pillar of plant antiviral defence or play a fundamental role in another process required for viral biology, such as cell-to-cell movement (Fig 1).

The C4 protein encoded by TYLCV has also been shown to partially relocalize from the plasma membrane to chloroplasts during infection. In chloroplasts, C4 interacts with CALCIUM SENSING RECEPTOR (CAS), described as a negative regulator of salicylic acid (SA)-dependent defences [16,17] (Fig 1). C4 suppresses the downstream activation of SA responses, although whether this activity relies on the physical interaction with CAS remains to be determined. CAS, nevertheless, has also been described as convergently targeted by effector proteins produced by pathogens belonging to different domains of life (namely HopAU1 from the bacterium *Pseudomonas syringae* pv. *actinidiae* and SSITL from the fungus *Sclerotinia sclerotiorum* [18,19]), which points to a broad role in plant-pathogen interactions (Fig 1).

A third function of C4 from TYLCV is to trigger the development of symptoms associated to the viral infection. Symptom development is accomplished through the co-option of members of the plant-specific RCC1-like domain-containing (RLD) protein family, involved in the establishment of cell polarity, through their recruitment to the plasma membrane [20–22]. While the roles of C4 in the suppression of plant defence mechanisms outlined above might have identified relevant players in plant-virus or plant-pathogen interactions, the mechanism underlying its manipulation of plant development highlights the systemic impact of potentially manipulating cell polarity in the single cell type TYLCV is restricted to, i.e., phloem companion cells.

**Fig 2:**
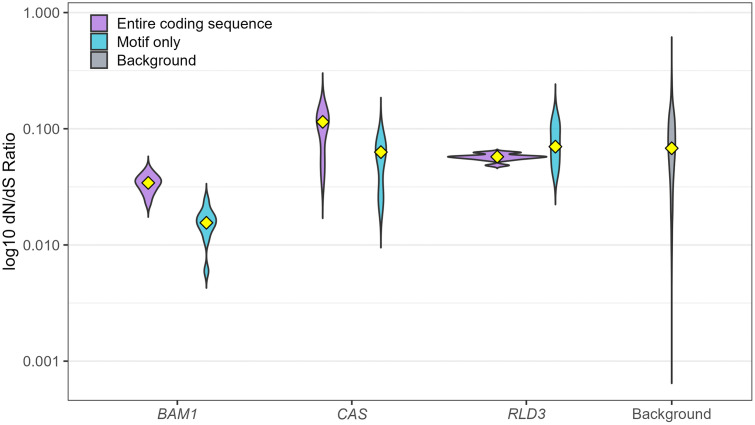
C4 target proteins tend to be more conserved than average. Violin plots summarize the distribution of codon-level dN/dS ratios for three *Arabidopsis thaliana* genes encoding proteins interacting with the C4 protein from tomato yellow leaf curl virus (TYLCV): *BAM1* (AT5G65700.1), *CAS* (AT5G23060.1), and *RLD3* (AT5G19420.2). For each gene, two categories are shown: the full-length coding region (“Entire coding sequence”) and a curated functional region (“Motif only”). “Motif” refers to the region of the protein proven to interact with C4 (amino acids 695–1009 in BAM1 from tomato; amino acids 231–387 in CAS from *A. thaliana*; amino acids 1030–1086 in RLD3 from *A. thaliana*). A genomic background distribution is shown for comparison. Yellow diamonds denote category means. Lower log10 dN/dS values indicate stronger purifying selection and greater conservation. The following species were used to identify orthologues of the genes of interest: *Beta vulgaris*, *Triticum Aestivum*, *Hordeum vulgare*, *Lotus japonicus*, *Nicotiana benthamiana*, *Phaseolus vulgaris*, *Solanum lycopersicum*, *Zea mays*, and *Manihot esculenta*.

C4, as suggested for viral proteins in general, also seems to target particularly conserved proteins or protein domains. Previous comparative genomics studies indicate that proteins targeted by viral effectors are frequently under strong purifying selection, showing very few amino-acid–changing mutations relative to silent ones (dN/dS), an indicator of selective pressure on protein-coding genes [23] commonly observed at interfaces of viral interactions [4]. Following this rationale, we evaluated the evolutionary conservation of the C4-interacting proteins BAM1, CAS, and RLD3 using an established workflow [4] to calculate their dN/dS values in comparison to their orthologs in nine plant species (see Fig 2). The results highlight BAM1 as particularly conserved across these plant species, showing a level of evolutionary constraint significantly stronger than the genetic background, as defined by the conservation values of a set of 200 random *Arabidopsis thaliana* genes.

When the interaction motifs previously shown to bind the geminiviral C4 protein were examined specifically, even stronger constraints became apparent: the BAM1 sequence encoding the C4-interacting motif displayed almost no variation across species, a pattern closely resembling that of CAS (Fig 2). These findings support previous work that viral interaction sites are subject to intense evolutionary constraint, reinforcing the broader notion that viral targets tend to be more conserved than average [4]. Interestingly, high conservation is detected for proteins convergently targeted by other pathogens (BAM1, CAS), where convergent targeting suggests a potential role in defence or other fundamental aspects of plant-pathogen interactions; this is not the case for RLD proteins, which are so far only known to interact with C4 and not influence viral accumulation, but only symptom development [20]. In this context, we propose that BAM1 and CAS might hold promise as potential targets for crop protection strategies, as would other plant proteins identified as interactors of diverse pathogen-encoded effectors [5].

## Some final considerations

Given the evolutionary pressures that shape virus-encoded proteins and their integration into host protein-protein interaction networks, studies of viral proteins can inform our understanding of infection and host biology beyond their immediate roles in virulence and pathogenesis; in this sense, viruses can be regarded as exceptional functional genomic tools. Inferring how viruses rewire cellular systems could not only illuminate how the infection is established and effected, and therefore uncover potential strategies for antiviral intervention, but also identify core cellular processes indispensable for maintaining life.
